# Optical Absorption Spectra and Electronic Properties of Symmetric and Asymmetric Squaraine Dyes for Use in DSSC Solar Cells: DFT and TD-DFT Studies

**DOI:** 10.3390/ijms17040487

**Published:** 2016-04-01

**Authors:** Reda M. El-Shishtawy, Shaaban A. Elroby, Abdullah M. Asiri, Klaus Müllen

**Affiliations:** 1 Chemistry Department, Faculty of Science, King Abdulaziz University, Jeddah B.O. 208203, Saudi Arabia; aasiri2@gmail.com; 2 Chemistry Department, Faculty of Science, Beni-Suef University, Beni-Suef 6251, Egypt; 3 Max-Planck Institute for Polymer Research, Ackermannweg 10, Mainz 55128, Germany; muellen@mpip-mainz.mpg.de

**Keywords:** squaraine dyes, TD-DFT, electron transfer, optical properties, HOMO-LUMO gap

## Abstract

The electronic absorption spectra, ground-state geometries and electronic structures of symmetric and asymmetric squaraine dyes (SQD1–SQD4) were investigated using density functional theory (DFT) and time-dependent (TD-DFT) density functional theory at the B3LYP/6-311++G** level. The calculated ground-state geometries reveal pronounced conjugation in these dyes. Long-range corrected time dependent density functionals Perdew, Burke and Ernzerhof (PBE, PBE1PBE (PBE0)), and the exchange functional of Tao, Perdew, Staroverov, and Scuseria (TPSSh) with 6-311++G** basis set were employed to examine optical absorption properties. In an extensive comparison between the optical data and DFT benchmark calculations, the BEP functional with 6-311++G** basis set was found to be the most appropriate in describing the electronic absorption spectra. The calculated energy values of lowest unoccupied molecular orbitals (LUMO) were 3.41, 3.19, 3.38 and 3.23 eV for SQD1, SQD2, SQD3, and SQD4, respectively. These values lie above the LUMO energy (−4.26 eV) of the conduction band of TiO_2_ nanoparticles indicating possible electron injection from the excited dyes to the conduction band of the TiO_2_ in dye-sensitized solar cells (DSSCs). Also, aromaticity computation for these dyes are in good agreement with the data obtained optically and geometrically with SQD4 as the highest aromatic structure. Based on the optimized molecular geometries, relative positions of the frontier orbitals, and the absorption maxima, we propose that these dyes are suitable components of photovoltaic DSSC devices.

## 1. Introduction

Dye-sensitized solar cells (DSSCs) represent one of the most promising approaches for the direct conversion of sun light to electricity at high efficiency with low cost [1,2,3,4,5]. DSSCs utilize sensitizing dyes adsorbed on the surface of TiO_2_ nanoparticles. The dye plays a vital role during absorption of light by which the excited electrons are injected into the TiO_2_ conduction band and travel to reach the counter-electrode. Squaraines are very attractive for such applications because they possess high extinction coefficients, inherent stability, and intense absorption in the far-red/near-Infrared (NIR) region. Several researchers have investigated squaraines as sensitizers for large-band gap oxide semiconductors [6,7,8,9,10,11,12,13]. The performance of DSSCs depends upon many factors such as the absorption efficiency of the sensitizing dye for the solar light spectrum. The electron transfer and separation of charge play important roles in the performance of DSSC. The electron transfer occurs between the highest occupied molecular orbital (HOMO) and lowest unoccupied molecular orbital (LUMO) of dyes and conduction band of TiO_2_. In view of these factors, which are related to ground and excited states, it is important to explore the electronic structures of both ground and excited states of the sensitizing dye molecules. Their electronic structures have been investigated using density functional theory (DFT), which has emerged as a reliable standard tool for theoretical treatment of organic dyes. In this regard, time-dependent (TD-DFT) calculations have been employed for studying the structures and absorption spectra of dye sensitizers for DSSCs [14,15,16,17,18,19,20,21,22,23]. In the present work, it was hypothesized that a good sensitizer molecule for DSSC which has one and/or two carboxyl anchoring groups needs to absorb in the NIR for a better light harvesting. Also, the presence of bulky alkyl groups in the sensitizer would be beneficial to prevent dye aggregation. To test the impact of these three parameters on the electronic properties the four dyes SQD1–SQD4 were designed. Furthermore, TD-DFT calculations were used to investigate the electronic absorption spectra, both, in the gas phase and in solvents of different polarity. Also, the energies of frontier molecular orbitals (FMO) of the studied dyes were evaluated to understand the electron transfer and charge separation mechanism.

## 2. Results and Discussion

**Scheme 1:**
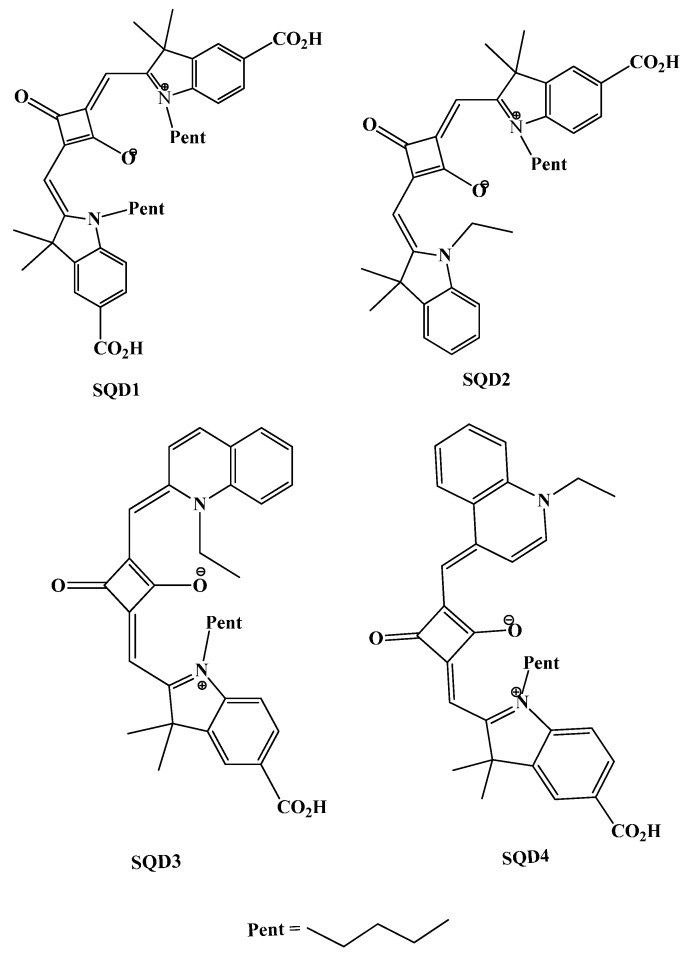
Chemical structures of the studied squaraine dyes.

SQD1–SQD4 share a squaric-indoline-carboxylic moiety as acceptor and differ in donors. Only SQD1 is a symmetric molecule and this structural difference was monitored via their optical, geometrical and electronic properties. Further, we have added different alkyl groups in different positions to reduce dye aggregation (Scheme 1).

### 2.1. Molecular Geometries

**Figure 1:**
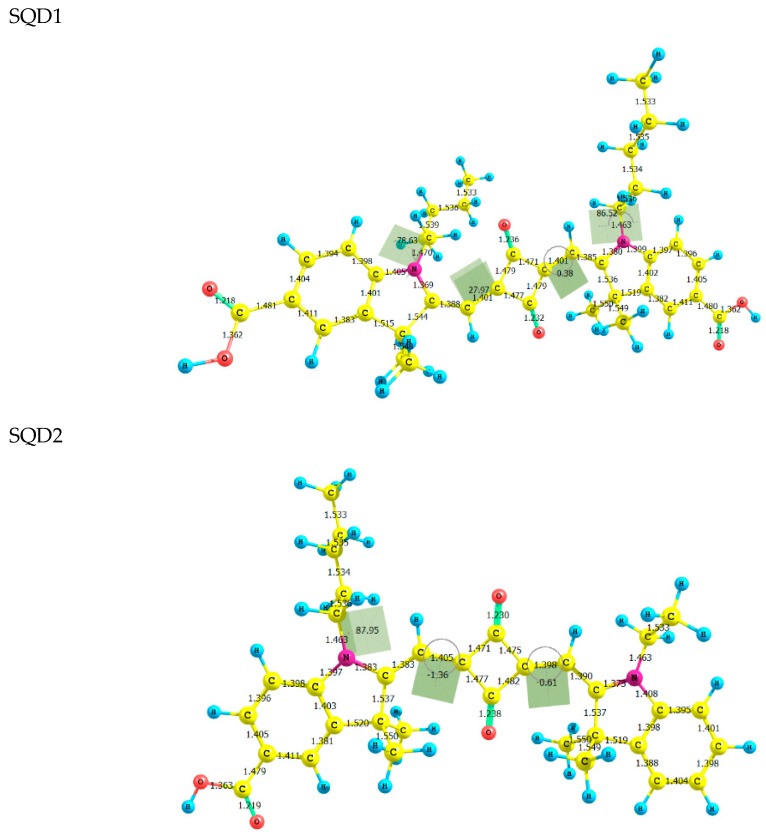

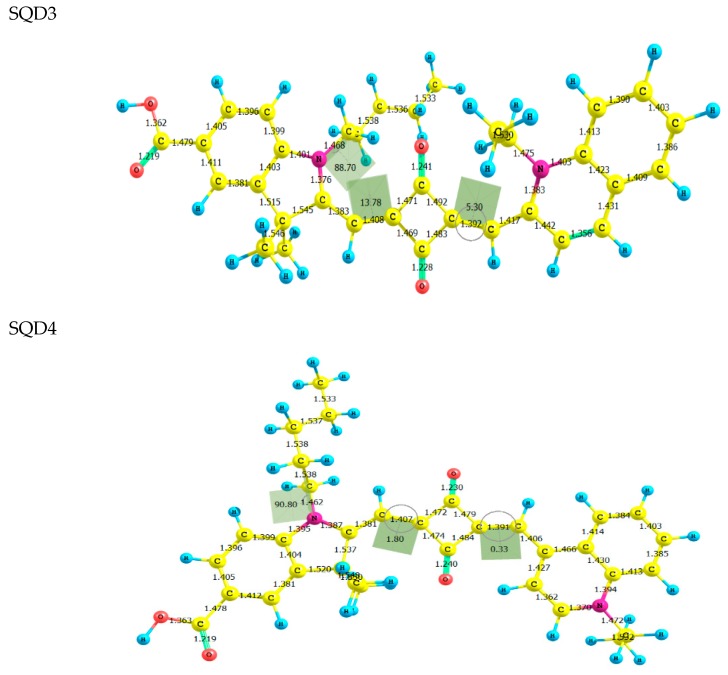
Optimized geometries and selected bond lengths (Å) and dihedral angles (°) of dyes SQD1-SQD4 using B3LYP/6-311++G** level of theory. Green color and rings indicate dihedral angles.

The optimized molecular geometries of SQD1–SQD4 in the ground state are shown in Figure 1. The squaric rings of all dyes appear almost planar, in contrast to the fact that squaric acid itself has a bent structure. For example, in the dye SQD1, the dihedral angel of the squaric ring with one indoline moiety is about 28 and 0.38° with the other indoline. These planar structures enhanced the aromatic character of the heterocyclic ring, and thus increasing the degree of electronic resonance between donor and acceptor moieties. On the other hand, pentyl groups present in all molecules result in a dihedral angels of 79°–91° as shown in Figure 1. In previous studies dyes with non-planar conformation were found to suppress molecular aggregation and reduce the rate of internal charge recombination, thus improving cell efficiency [9]. Therefore, it is anticipated that this large dihedral angles caused by the presence of pentyl groups in SQD1–SQD4 would lower the aggregation of dye molecules. The C–C bond lengths in squaric rings, heterocyclic moieties in both sides and the connecting bonds between them range from 1.37–1.48 Å, which are shorter than a C–C single bond (1.54 Å), but longer than a C=C double bond (1.34 Å, indicating pronounced resonance structures for all dyes). The replacement of indoline moieties shown in SQD1 and SQD2 with quinoline present in SQD3 and SQD4 increased the C–C bond lengths that connect them with squaric ring from 1.39 to 1.41 Å, respectively. This result is nicely correlated with the fact that the indoline moiety is non-aromatic, whereas quinoline one is aromatic.

### 2.2. Electronic Absorption Spectra

**Figure 2:**
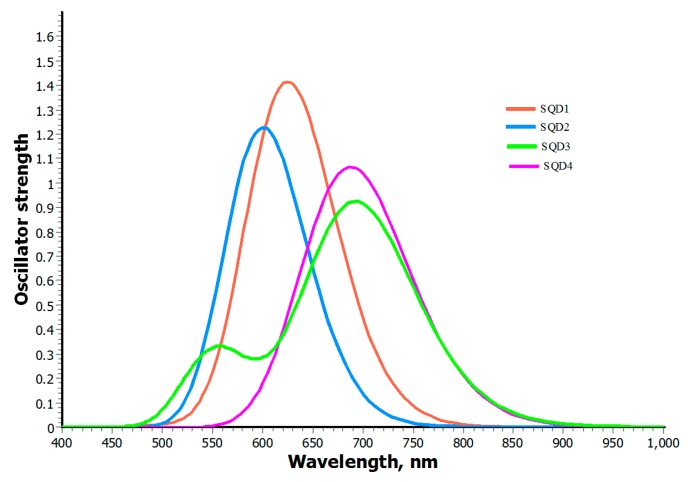
The UV-visible absorption spectra of SQD1–SQD4 calculated using PBE/6-311++G** level of theory in the gas phase.

**Figure 3:**
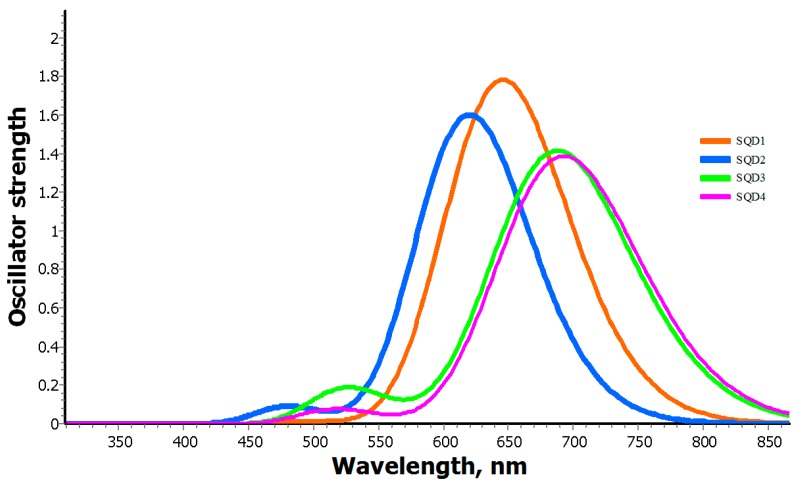
The UV-visible absorption spectra of SQD1–SQD4 dyes calculated using PBE/6-311++G** level of theory in methanol.

**Scheme 2:**
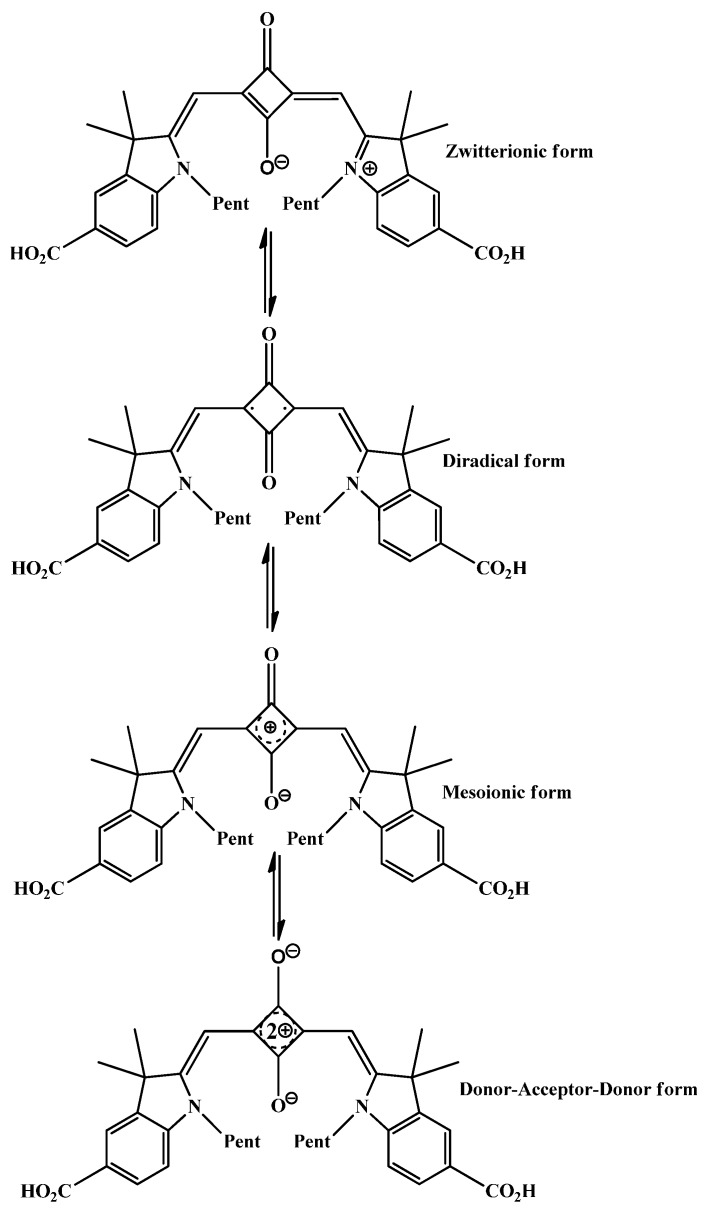
Resonance structures of SQD1.

The simulated absorption spectra for SQD2 and SQD4 as examples using different TD-DFT functionals in the gas phase and for SQD4 using PBE/6-311++G** in different solvents are displayed in Figures S1 and S2, respectively. The PBE/6-311++G** was found to have produced spectra in good agreement with experimental data. Applying this method in calculating the spectra of SQD1–SQD4 in the gas phase and methanol resulted comparable data shown in Figure 2 and Figure 3, respectively. The computed UV-visible absorption data of SQD1–SQD4 in the gas phase and those in solutions are collected in Table 1 and Table 2. In general, the experimental values of λ_max_ of these dyes show the same trend as the calculated ones. For example, the absorptions in methanol solution were deviated by 13–58 nm. A similar deviation between calculated and observed wavelengths for other squaraine dyes has also been observed and attributed to the approximations inherent to the TD-DFT calculation and together with the diradical nature of such molecules [24,25,26]. A recent study has revealed that the absorptions of squaraine dyes are red-shifted to NIR by virtue of a large contribution of a diradical character [24]. Thus, a resonance contribution to SQD1, for example, can be represented in Scheme 2. Therefore, it is anticipated that the blue shift observed for SQD2 compared with SQD1 can be rationalized based on the extent of diradical character in SQD1 but not to the strength of the electron donating group. Expectedly, SQD3 and SQD4 have λ_max_ values more red-shifted in theoretical and experimental than those of SQD1 and SQD2 owing to the extended conjugation caused by the quinoline moiety in both SQD3 and SQD4.

**Figure 4:**
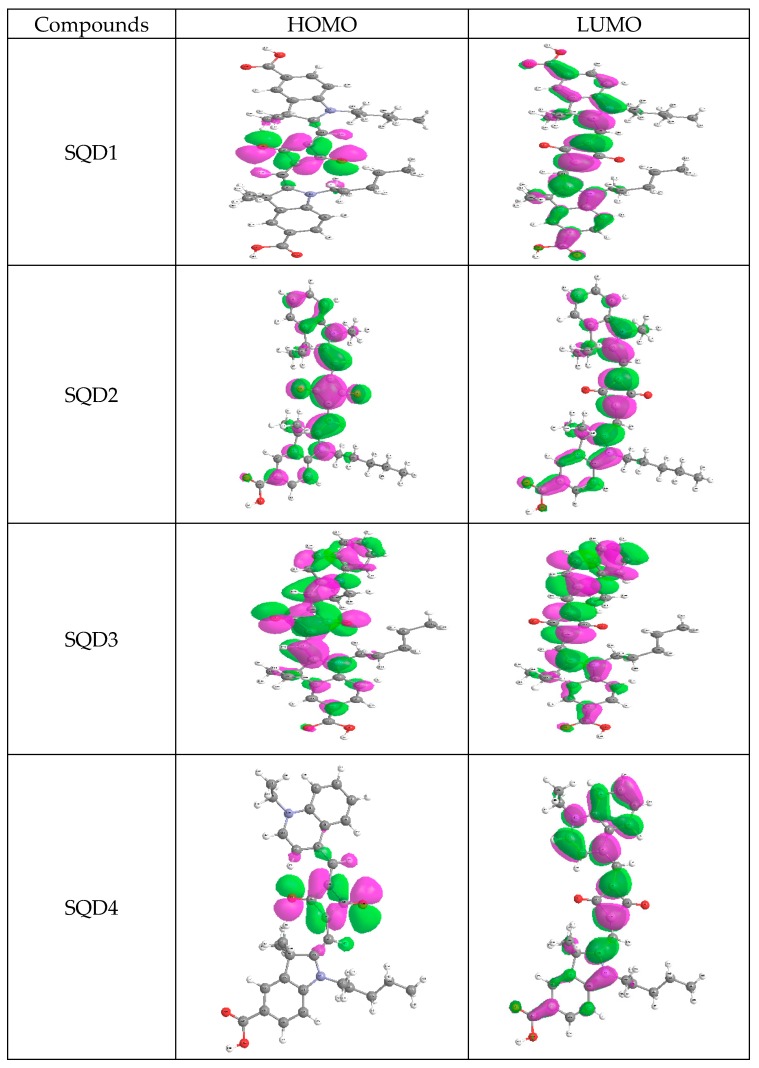
Schematic diagram of natural transition orbitals (NTOs) of the studied dyes calculated at the PBE/6-311++G** level of theory. The surfaces are generated with an isovalue at 0.02.

For all dyes, the λ_max_ is assigned to be mainly due to a HOMO→LUMO transition, whereas the second weak absorption corresponds to HOMO-LUMO + 1 or HOMO-1→LUMO transitions. These results in Table 2 show that all studied dyes share an intense π→π* absorption, where the electron density is mainly transferred from the HOMO to the LUMO (> 65%). Table 1 presents the electronic energy levels and gap energy using PBE/6-311++G** level of theory. The HOMO and LUMO energies of SQD1, SQD2, SQD3 and SQD4 are −4.68, −4.50, −4.47, −4.29 and −3.41, −3.19, −3.38, −3.22 eV, respectively. These values indicate that the HOMO energy in dye SQD4 is highest one due to the presence of the para-quinoline group. The E_LUMO_ values for all dyes are located above the conduction band edge of TiO_2_ (−4.26 eV) [27]. The relative matching of electronic levels of sensitizers would lead to energetically favorable electron injection as well as regeneration of oxidized dye during DSSC operation. Figure 4 shows the calculated electronic energies of frontier orbitals and corresponding surface density plots. The natural transition orbitals (NTO) of the studied dyes are presented in Figure 4. As demonstrated in Figure 4, the electron distributions of the HOMO orbitals of the dyes were mostly localized over the squaric ring, whereas those of the LUMO orbitals were mainly localized in the indoline and its attached carboxylic group. Furthermore, the results indicated that the HOMO–LUMO excitation induced by light irradiation can effectively move the electron distribution from the squaric-indoline moiety to the carboxylic anchoring group leading to an electron injection if the carboxyl group is attached to TiO_2_ semiconductor. The decrease in the HOMO–LUMO gap could be attributed to the increase in π-electron density of the molecule, which leads to an increase in diradical character and bathochromic shift.

### 2.3. Aromaticcity Computation

Further insight into the chemical reactivity analysis of SQD1–SQD4 in relation to their electronegativity, chemical hardness and their aromaticity difference would assess the suitability of these dyes for use in DSSC solar cells.

The electronegativity based on atoms before being bonded to form a molecule is calculated by the following equation [28,29,30,31]:

(1) $$\chi_{\text{AIM}}=\frac{n_{\text{AIM}}}{\sum_{A}\frac{n_{A}}{\chi_{A}}}$$

where AIM refers to atoms in a molecule, $n_{\text{AIM}}$ is the number of atoms in a molecule, $\underset{A}{\sum }\frac{n_{A}}{\chi_{A}}$ is the summation of ratios of number of atoms for each a-species divided by its corresponding atomic electronegativity. The molecular electronegativity in the post-bonding stage of a molecule is calculated by the following equation:

(2) $$\chi_{\text{MOL}}≅-\frac{E_{\text{HOMO }\left(1\right)}+E_{\text{LUMU }\left(1\right)}}{2}$$

Combining both Equations (1) and (2) gives the aromaticity index-based electronegativity (*A*_EL_):

(3) $$A_{\text{EL}}=\frac{\eta_{\text{AIM}}}{\chi_{\text{MOL}}}$$

Similarly, the chemical hardness ($\eta_{\text{AIM}})$ per-ponding and after bonding ($\chi_{\text{MOL}})$ in a molecule are given by Equations (4) and (5), respectively:

(4) $$\eta_{\text{AIM}}=\frac{n_{\text{AIM}}}{\sum_{A\;}\frac{n_{A}}{\eta_{A}}}$$

where AIM refers to atoms in a molecule, $n_{\text{AIM}}$ is the number of atoms in a molecule, $\underset{A}{\sum }\frac{n_{A}}{\eta_{A}}$ is the summation of ratios of number of atoms for each A-species divided by its corresponding atomic hardness.

(5) $$\eta_{\text{MOL}}≅E_{\text{LUMU}}-E_{\text{HOMO}}$$

Combining both Equations (4) and (5) gives the aromaticity index-based chemical hardness (*A*_Hard_):

(6) $$A_{\text{Hard}}=\frac{\eta_{\text{AIM}}}{\eta_{\text{MOL}}}$$

The data in Table 3 indicates that the aromaticity indices based on electronegativity and chemical hardness are in good agreement with those obtained optically and geometrically presented above.

## 3. Materials and Methods

### 3.1. UV–Visible Absorption Spectra of Squaraine Dyes (SQD1–SQD4)

UV-visible absorption spectra of SQD1–SQ4 were measured in three different solvents (tetrahydrofuran, dichloromethane and methanol) so as to determine the maximum wavelength of absorption. UV-visible absorption spectra were recorded with a Jasco V560 spectrophotometer (Jasco international Co., Ltd., Tokyo, Japan).

### 3.2. Computational Methods

All calculations were performed using the Gaussian 09W [32] program package. B3LYP/6-311++G** level of theory was employed using Becke’s three parameter hybrids function combined with the Lee–Yang–Parr correlation function (B3LYP) [33,34,35,36] to predict the molecular geometry and electronic transition for moderately large molecules. B3LYP/6-311+G** frequency analysis calculations were performed to characterize the stationary points as the minima. HOMO–LUMO energies, absorption wavelengths (λ_max_) and oscillator strengths (f) were calculated using TD-DFT with B3LYP/6-311++G** [37,38] level based on optimized structures in the gas phase. Moreover, three density functionals, namely, the TPSSh [39,40,41,42], PBE, and PBE1PBE (PBE0) [43] with the 6-311++G** basis set have been evaluated. Long-range correction has solved the underestimations of charge transfer excitation energies and oscillator strengths in time-dependent Kohn–Sham calculations and has clearly improved poor optical response properties [44]. The UV-vis spectra of dyes in different solvents were calculated by TD-PBE/6-311++G** level. Solvation effects were introduced by the SCRF method, via the conductor polarizable continuum model (CPCM) [45,46].

## 4. Conclusions

Electronic structures and geometries of the ground-state of symmetric and asymmetric squaraine dyes SQ1–SQ4 in the gas phase were investigated by B3LYP/6-311++G** level of theory. The calculated geometric data indicate strong conjugation effects in these dyes, which is beneficial for the optical properties. UV-visible spectra and frontier molecular orbitals were studied by different TD-DFT functionals, namely: PBE, PBE1PBE (PBE0), and TSSPh with 6-311++G** basis sets in the gas phase and different polar solvents. The first optically allowed electronic transitions of SQ1–SQ4 at PBE/6-311++G** level of theory is predicted the contribution of the HOMO-LUMO transition at 625, 600, 693, and 687 nm, respectively. The quinoline moiety may be a better unit for red-shifting the absorption of squaraine dye as shown in SQD4 spectra.

The red shift of absorptions of SQD2 compared with SQD1 might be attributed to the large contribution of a diradical character in SQD2. From frontier molecular orbital calculations, the E_LUMO_ are −3.41, −3.19, −3.38, and −3.23 eV for SQ1–SQ4, respectively. These values lie above the LUMO energy (−4.26 eV) of the conduction band of TiO_2_ nanoparticles indicating possible electron injection from the LUMO of the dyes to the conduction band of the TiO_2_ in DSSCs. Absorption bands of SQD1–SQD4 could be easily extended into NIR region by straightforward structural modification, which closely match the spectral response of sun light. 

## Figures and Tables

**Table 1:** Absorption wavelength (nm), molecular orbital contribution, energy level of HOMO, LUMO and gap energy and oscillator strength calculated by using PBE/6-311++G** for squaraine studied dyes in the gas phase.

Compounds	Wave Length (nm)	Oscillator Strength (f)	MO Contribution	MO Coeff.	E_HOMO_ eV	E_LUMO_ eV	Gap Energy = E_LUMO_−E_HOMO_ eV
SQD1	624.86	1.416	HOMO-LUMO	70%	−4.68	−3.41	1.27
	512.53	0.012	HOMO-1-LUMO	68%			
SQD2	807.79	0.0005	HOMO-1-LUMO	70%	−4.50	−3.19	1.31
	600.35	1.2269	HOMO-LUMO	69%			
	504.43	0.0001	HOMO-2-LUMO	68%			
SQD3	864.25	0.002	HOMO-1-LUMO	70%	−4.47	−3.38	1.09
	692.73	0.924	HOMO-LUMO	68%			
	554.05	0.324	HOMO-LUMO+1	66%			
SQD4	687	1.06	HOMO-LUMO	70%	−4.29	−3.23	1.06
	545.92	0.00	HOMO-1.LUMO	71%			

MO: Molecular Orbital.

**Table 2:** Experimental and theoretical absorption wavelength (nm) by using PBE/6-311++G** for squaraine studied dyes in different solvents.

Compounds	Tetrahydrofuran				Dichloromethane				Methanol			
	Cal.	f	Exp.	Dev.EXP	Cal.	f	Exp.	Dev.EXP	Cal.	f	Exp.	Dev.EXP
SQD1	652	1.81	691	−39	642	1.74	685	−43	655	1.77	677	−22
SQD2	627	1.56	679	−52	620	1.58	677	−57	629	1.61	668	−58
SQD3	702	1.42	729	−27	688	1.41	717	−29	711	1.39	698	−13
SQD4	705	1.23	817	−112	695	1.06	785	−90	705	0.92	747	−42

Cal.: Calculated; Exp.: Experimental; f: Oscillator Strength.

**Table 3:** The molecular electronegativity and chemical hardness, along the quantum compactness aromaticity A_EL_ and A_Hard_ indices for studied dyes at PBE/6-311++G** level of theory. all energetic values in electronvolts (eV).

Compounds	*χ*_ΑΙΜ_	*η*_ΑΙΜ_	*χ*_MOL_	*η*_MOL_	*A*_EL_	*A*_Hard_
SQD1	6.769	5.741	0.635	8.10	10.660	0.709
SQD2	6.750	5.727	0.655	7.690	10.306	0.745
SQD3	6.735	5.703	0.545	7.850	12.359	0.726
SQD4	6.735	5.703	0.53	7.520	12.708	0.758

## Supplementary Materials

Supplementary materials can be found at http://www.mdpi.com/1422-0067/17/4/487/s1.
