# Supplementary material for: Optical Absorption Spectra and Electronic Properties of Symmetric and Asymmetric Squaraine Dyes for Use in DSSC Solar Cells: DFT and TD-DFT Studies
Source: Int J Mol Sci. 2016 Apr 1;17(4):487. doi: 10.3390/ijms17040487 (PMC4848943; doi:10.3390/ijms17040487)
Supplement: Supplementary file 1 [file ijms-17-00487-s001.pdf]

Supplementary Materials: Optical Absorption Spectra and Electronic Properties of Symmetric and Asymmetric Squaraine Dyes for Use in DSSC Solar Cells: DFT and TD-DFT Studies

Reda M. El-Shishtawy, Shaaban A. Elroby, Abdullah M. Asiri and Klaus Müllen

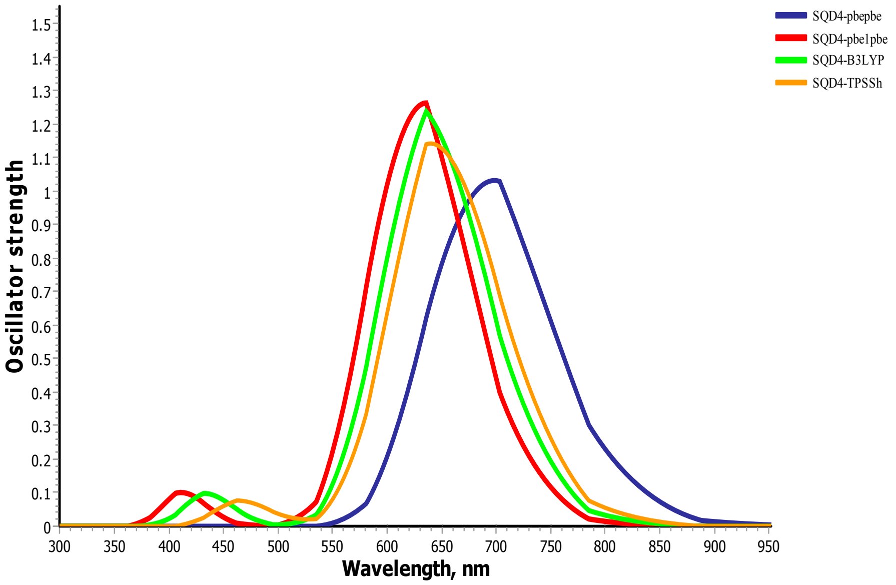

Figure S1. The UV-visible absorption spectra of SQD2 and SQD4 dyes calculated at different functionals with 6-311++G** basis set in the gas phase.

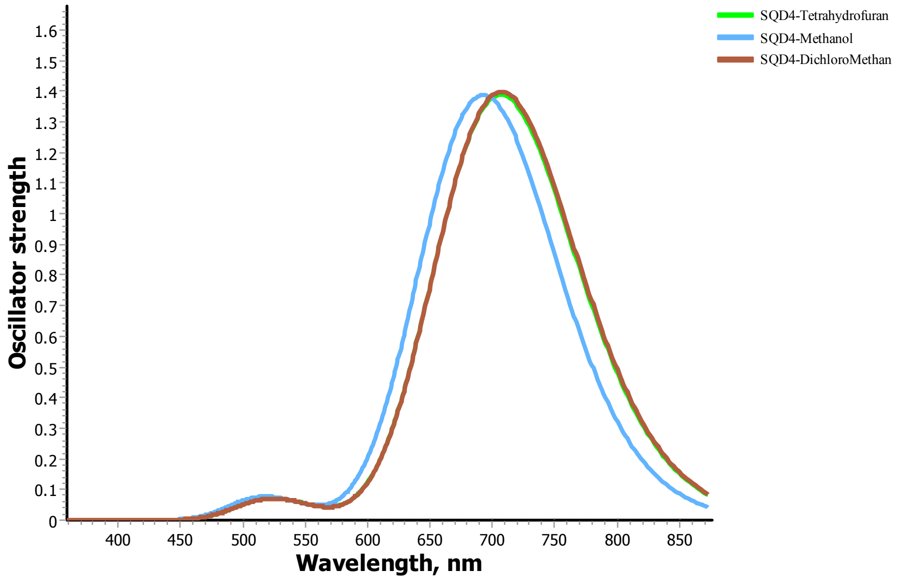

Figure S2. The UV-visible absorption spectra of SQD4 dyes calculated using PBEPBE/6-311++G** in different solvents.
